# Artificial Intelligence versus Doctors' Intelligence: A Glance on Machine Learning Benefaction in Electrocardiography

**DOI:** 10.15190/d.2017.6

**Published:** 2017-09-30

**Authors:** Victor Ponomariov, Liviu Chirila, Florentina-Mihaela Apipie, Raffaele Abate, Mihaela Rusu, Zhuojun Wu, Elisa A. Liehn, Ilie Bucur

**Affiliations:** Institute for Molecular Cardiovascular Research (IMCAR), RWTH Aachen University, Germany; Department of Cardiology, Pulmonology, Angiology and Intensive Care, University Hospital, RWTH Aachen University, Germany; ECUORE LTD, London, England; Applied Systems srl, Craiova, Romania; Faculty of Economic and Business Administration, Doctoral School of Economics, University of Craiova, Romania; School of Medicine, University of Catania, Italy; Institute for Molecular Cardiovascular Research, University Hospital, RWTH Aachen, Germany; IZKF, Aachen, RWTH Aachen, Germany; Human Genetic Laboratory, University of Medicine and Pharmacy, Craiova, Romania; Applied Systems, Craiova, Romania

**Keywords:** machine learning, machine intelligence, algorithms, artificial intelligence, computational models, multiple processing layers, autonomic learning

## Abstract

Computational machine learning, especially self-enhancing algorithms, prove remarkable effectiveness in applications, including cardiovascular medicine. This review summarizes and cross-compares the current machine learning algorithms applied to electrocardiogram interpretation. In practice, continuous real-time monitoring of electrocardiograms is still difficult to realize. Furthermore, automated ECG interpretation by implementing specific artificial intelligence algorithms is even more challenging. By collecting large datasets from one individual, computational approaches can assure an efficient personalized treatment strategy, such as a correct prediction on patient-specific disease progression, therapeutic success rate and limitations of certain interventions, thus reducing the hospitalization costs and physicians’ workload. Clearly such aims can be achieved by a perfect symbiosis of a multidisciplinary team involving clinicians, researchers and computer scientists. Summarizing, continuous cross-examination between machine intelligence and human intelligence is a combination of precision, rationale and high-throughput scientific engine integrated into a challenging framework of big data science.

## SUMMARY

1. *Introduction*
2. *Basic aspects of machine learning*
3. *Basic aspects of ECG-machine learning*
4. *Detection of ECG components and disease-specific abnormalities in ECG*
5. *ECG prediction of cardiovascular events and detection of unknown patterns*
6. *Future considerations*
7. *Conclusion*

## **1. **Introduction

As an area of computational science, the machine learning subfield has at its core self-enhancing algorithms (**Table 1**), which are improved with experience, to give remarkable effectiveness in applications, including cardiovascular medicine.

**Table 1:** The main current machine learning algorithms applied to electrocardiogram interpretation.

Machine-learning algorithms	Type of learning	Method of extracting parameters	References
Fuzzy C-Means (FCM) clustering algorithm	Unsupervised	Mahalanobis-Taguchi System (MTS)	^1^
Artificial Neural Networks (ANN)	Supervised	Multi Resolution; Wavelet analysis; Descriptor Haar-like; Fast Fourier transform; Discrete Wavelet transform; Wavelet transform process	^2-21^
Support vector machine	Supervised	Stockwell transform; Bacteria foraging optimisation algorithm; Linear discriminant analysis; Wavelet transform	^7,12,13,21-27^
Random forests (RF)	Supervised	Wavelet packet entropy (WPE)	^21,22,28^
K-nearest neighbours	Supervised	Higher order statistics of wavelet packet decomposition	^13,29-31^
Logistic Regression	Supervised	-	^2,21^
Fuzzy Inference System	Supervised	Linear discriminant analysis, Wavelet transform	^21,23^
Naive Bayesian	Supervised	-	^2,29^
Gradient boosting machines	Supervised	-	^21^
Decision Trees	Supervised	-	^2^

In practice, continuous real-time monitoring of the electrocardiogram is, still difficult to realize, although such tasks can be addressed successfully using machine learning methods^32-35^. For instance, machine learning algorithms can be applied for automated interpretation of the ECG, which will immediately detect, classify, and report an arrhythmic event^5,36^. Additionally, algorithms can facilitate ECG reading while eliminating artefacts, filtering maternal ECG to detect fetal heart rate^37^ or extract heart rate variability^38^. Moreover, computational models can be fused into a hybrid system in order to achieve superior results^39,40^. The incorporation of machine learning algorithms can simultaneously process multiple risk factors, valorizing more nuanced relationships between those risk factors^41^.

Here, we aim to summarize and cross-compare the current machine learning algorithms applied to electrocardiogram interpretation, focusing on the main methods and essential aspects about putting them into practice. Nonetheless, our paper can serve as guidance for the newcomers into this area of research, either as a computer scientist, biomedical researcher or physician, as we present common difficulties of the automated ECG interpretation. We discuss and outline challenges on the way of implementing specific artificial intelligence algorithms, while avoiding trivial details and highlighting the achievements.

## **2. **Basic aspects of machine learning

“Machine learning” concept was introduced by A. Samuel in 1959 to describe the algorithms allowing the computers to make self-predictions when analyzing big sets of data^42^. These algorithms introduced specific pattern recognitions, thus the computers learn and adapt to perform specific tasks without being programmed. Independent of applied field, machine learning is able to process a huge amount of data and to automatically produce complex models to analyze them and to deliver very fast accurate and reproducible results. This is a very cheap way to identify opportunities or to avoid unknown risks.

In Medicine, machine learning should help physicians to make right diagnosis and to choose the right treatment for each patient, such called personalized medicine^43^. However, due to the limited availability of consistent healthcare data and differences in acquiring the data between the institutions^44^, the developed algorithms can make wrong predictions which can potentially, for example, taint otherwise safe drugs with bad reputation for a long time^45-48^. Therefore, machine learning in public health demands to be flexible, dynamic and regularly up-dated, which suppose a coordinate investment in global public health infrastructure^49^.

Machine learning algorithms find great potential in facilitating precision in diagnosis and prediction in cardiovascular medicine^50^, the main cause of morbidity and mortality in the world. Moreover, the collaborative learning using multidisciplinary approach allow for rapid developing and dissemination of the information, objectively identify and prioritize focused problems for guideline development^51^.

From all diagnostic methods in cardiovascular diseases, ECG is the most accessible and, therefore, an area of intense research. Despite of different pattern recognition algorithms applied in ECG signals recognition in the past decades, a valuable application (**Table 1**) has been not yet established, and future efforts should be directed to this field^7^.

## 3. Basic aspects of ECG-machine learning

**Figure 1:**
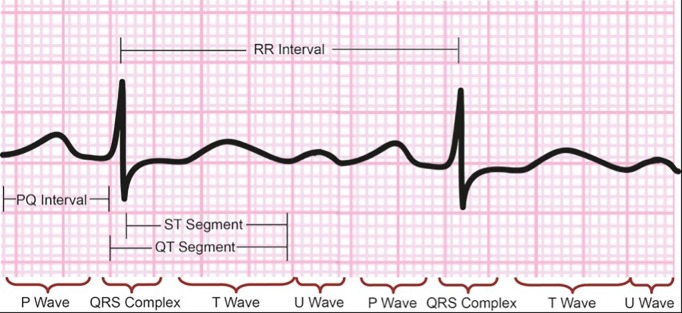
Normal ECG Signal

The electrical activity of the heart on ECG recording is represented by a time-series of waves (P, QRS complex, T, and U), intervals (PR, QT, RR), segments (ST, TP) (**Figure 1**). Detection of the components on the electrocardiogram requires the application of a classifying method, which acts in a discriminative manner based on the input features.

The first step in interpreting the ECG signal is to classify the signal as a normal or an abnormal heart activity. The detection of normal heart activity will stop further ECG signal interpretation, essential for minimizing the false results of ECG. Therefore, research is focused on different algorithms, developed to detect abnormal heart activity^2,3,5,6,22,41,52,53^.

Roughly, there are two machine learning approaches: (1) supervised or (2) unsupervised learning method^40^.

*Supervised* methods are taught with annotated examples from which the algorithm learns to recognize or predict patterns on unlabeled examples, whereas unsupervised methods learn to spot patterns in a data set without labels. A typical application of algorithms trained via supervised learning techniques is the classification of data in known categories. The data must be annotated by a human supervisor or through automatic collection^54^. Illustrative examples of this approach are found in fields ranging from handwritten digit recognition^55,56^ to the classification of cancerous cells^57^.

A supervised and inductive learning algorithm, VFI5, was used successfully in the diagnosis of cardiac arrhythmia from standard 12 lead ECG recordings^29^. Another supervised learning method used in the diagnosis of cardiac arrhythmia is *Probabilistic Neural Network* implemented by Gutiérrez-Gnecchi et al^4^. This platform was intended for on-line, real-time ambulatory operation and can classify eight heartbeat conditions. The results derived from confusion matrix tests yielded an overall on-line classiﬁcation accuracy of 92.746%.

On the other hand**, ***unsupervised* learning attempts the extraction of the most significant features of a non-annotated set of data: it is in fact a technique often used for denoising purposes, to extract not explicit correlations from distributions, to cluster data into groups sharing similar characteristics^54,58^.

Examples of tasks involving unsupervised learning models are: the extraction of fetal QRS complex from the maternal ECG^59^, the clustering of ECG beats in holter records^60^, the preprocessing of color-Doppler data^61^, the prediction of patients' future from electronic health records^62^.

Fuzzy C-Means (FCM) clustering algorithm improved with the attribute selection model based on Mahalanobis-Taguchi System (MTS) is an unsupervised learning method. It was implemented by Nur Al Hasan Haldar et al.^1^ on the arrhythmia dataset provided by MIT-HIB. The authors claim that their proposed work is *also suitable for mobile health monitoring* and well-suited for wireless body area network (WBAN) environment.

It is important to note that there is not a strict distinction between supervised and unsupervised learning algorithms^40,54^: quite often these two different methods overlap and are combined to perform classifications and regression tasks in big datasets, which require complex preliminary analysis in order to be successfully processed^63^.

Semi-supervised learning is in fact another approach exploiting the data decomposition and features extraction capabilities of unsupervised learning algorithms together with the high accuracies provided by the presence of a supervisor during the training process. Among the most used, Artificial Neural Networks allow the analysis of cardiovascular issues using both supervised and unsupervised learning methods.

Recent works in ECG classification^64^ use both unsupervised and supervised learning algorithms in order to achieve outstanding performances (**Table 1**).

As much as it would be desired in artificial intelligence, there is no general approach that can unrestrictedly handle any task. The selection of specific machine learning algorithms is dependent on the individual practical problem to be solved. More importantly, it is advisable to thoroughly explore and understand the tasks’ critical details. An appropriately selected method does not lead to success unless matched perfectly with the prior field-specific knowledge.

For example, consider morphological feature extraction step from an ECG trace. Some heart conditions can drastically change ECG components, thereby feature extraction step should respect all potential morphological variations.

**Figure 2:**
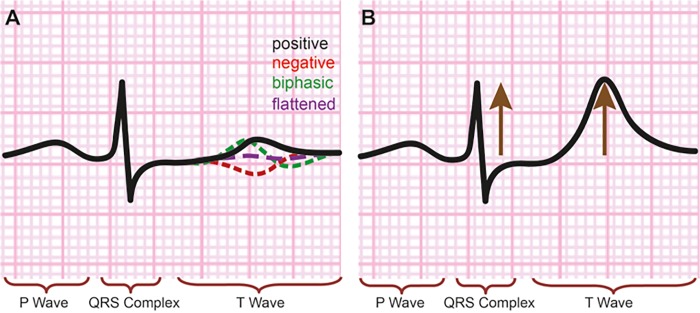
T Waves in normal ECG Signal. (A) Possible T Waves forms in pathological situations: positive (black), negative (red), biphasic (green) and flattened (violet). (B) Higher amplitude of T Wave (brawn arrow).

When looking at the ECG, the first wave which follows QRS complex is called T wave. T waves can present as a positive deflection, inverted (negative deflection), biphasic wave (half positive, half negative) or even become flattened during certain dynamic heart conditions (e.g. ischemia) (**Figure 2** [A]). While some algorithms can filter QRS complex^65^, concerns will be raised whether high peaked T waves (**Figure 2** [B]) can be discriminated from QRS complex when extracting all ECG components. For instance M. Vijayavanan et al. used an algorithm to calculate the heart rate based on that R wave within the QRS complex invariably which has the highest amplitude^66^. It can be anticipated that such a method can easily misinterpret, since there are examples when T waves have the highest amplitude (**Figure 2** [B]).

Therefore, the accuracy of automated processing ECG signals task is still an issue to be solved, which creates a large variability between all the existent programs that attempt to analyses ECG signals.

## 4. Detection of ECG components and disease-specific abnormalities in ECG

The detection of cardiac-specific abnormalities via ECG can be quite challenging, as the ECG patterns is highly dependent on the time point of the ECG recording and can change over time when the patient is symptomatic or asymptomatic^65^. For examples, during the active ischemia phase of acute coronary syndrome, the ECG can reliably track changes such as ST-segment elevation, reduction of the S wave, increase of T wave amplitude and QRS distortion. However, ECG shows only minimal changes during reperfusion stage. As a result, ECG analysis by itself can lead to misdiagnosis. Serial ECG recording and prior recording can greatly increase the detection threshold in combination with clinical manifestations. Moreover, different diseases might present similar ECG abnormalities such as viral myocarditis mimicking acute myocardial infarction^65^, making a disease-specific detection via ECG even more difficult.

A method to classify arrhythmia by eight heartbeat conditions: normal sinus rhythm (N), auricular fibrillation (AF), premature atrial contraction (PAC), left bundle branch block (LBBB), right bundle branch block (RBBB), premature ventricular contraction (PVC), sinoauricular heart block (SHB) and supraventricular tachycardia (SVT) is implemented on a platform intended for on-line, real-time ambulatory operation. The algorithm uses a wavelet transform process based on quadratic wavelets for identifying individual ECG waves^4^.

## 5. ECG prediction of cardiovascular events and detection of unknown patterns

Extensive clinical evidence provided by scientists is the most important instrument for identifying health risks. Using empirical data and autonomic learning, we can expand our horizons and nourish our fundamental knowledge with relevant hypotheses. Disease prevention is an action of developing early detection strategies, which aim to protect and improve global health. The exploration of unknown patterns and its assembly into prediction systems for cardiovascular events are essential sources for innovation. For example, there are reports of low sensitivity of the vital signs as predictors of clinical outcome in critically ill emergency department patients^67^. Meanwhile machine learning-based methods for variable selection and further prediction of cardiac events showed promising results^67^.

In concrete examples, X. Tang and L. Shu^8^ used a rough sets (RS) and a Quantum Neural Network (QNN) to recognize ECG signals, classify and obtain fast and realistic forecast of cardiological events. In one of the most encouraging recently work, Stephen F. Weng et al.^21^ predicted first cardiovascular event over 10-years with four machine-learning algorithms: random forest, logistic regression, gradient boosting an neural networks compared to an established algorithm (American College of Cardiology guidelines). In the study, they used routine clinical data of 378,256 patients from UK family practices, free from cardiovascular disease at outset. Neural networks was the highest achieving algorithm, which predicted 4,998/7,404 cases (sensitivity 67.5%,) and 53,458/75,585 non-cases (specificity 70.7%), therefore correctly predicting 355 more patients (+7.6%) who developed cardiovascular disease compared to the established algorithm.

## **6. **Future considerations

Personalized medicine is about applying the appropriate treatment and avoiding unnecessary interventions, and consequently providing accurate and cost-efficient care. Through grouping patients based on their genetic background, past medical history, current health state, it is sought to predict the optimal medical decision. Moreover, through the collection of large data sets from single individuals, predictions on patient-specific disease progression, therapeutic success rate and limitations of certain interventions can be made.

In light of this, data analysis is crucial; thus, the role of artificial intelligence is irreplaceable. For example, Badilini et al. trained a model which is able to detect drug-specific changes of the electrocardiogram, based on the wave’s morphology^67^. In this study, they have used Sotalol, which can cause Torsades de pointes, and by training algorithms to recognize early features, automated prediction in the early stages of disease development can be spotted and subsequent alarms activated. Indeed, subtle drug-induced ECG changes are difficult to recognize and machine aid in healthcare is a justifiable research direction to improve clinical decision. Computational approaches are suitable for assessing the heterogeneity of the diseases’ natural history. Extraction of the essential disease-features is the foundation of personalized and precision medicine.

The results suggest that the method and prototype presented may be suitable for implementation on wearable sensing applications auxiliary for on-line, real-time diagnosis^4^.

The world-wide burden of chronic diseases is forcing healthcare providers to implement new strategies for accurate patient monitoring, meanwhile reducing hospitalization costs and physicians’ workload. Under these circumstances, technological advancements are making their print on providing medical services.

Recent meta-analysis showed a near fifty percent drop for the time-to-treatment of the acute myocardial infarction when using pre-hospital triage with telemedicine^67^.

In the field of telecardiology, efforts are made towards automated real-time ECG diagnosis with wearable devices^67^. In addition, to obtain more advantages of monitoring devices, these devices are compatible for integrations into a platform for automated development of databases. Eventually, stored data can be computationally embedded into a cycle of self-teaching algorithms and serve as a source of accessible raw material for research purposes.

Finally, a reasonable future trend is towards a 12-lead interpretation with real-time diagnosis and continuous algorithm improvement.

## **7. **Conclusion

Clearly, artificial intelligence application for biomedical signal processing cannot be tackled if there is a lack of perfect symbiosis of a multidisciplinary team involving clinicians, researchers and computer scientists.

Summarizing, continuous cross-examination between machine intelligence and human intelligence is a combination of precision, rationale and high-throughput scientific engine integrated into a challenging framework of big data science

- **Autonomic learning using empirical data can expand our horizons and nourish background knowledge with relevant hypotheses.**
- **Computational approaches are suitable to assess the heterogeneity of diseases**
- **Extraction of the essential disease features by computational algorithms is the foundation of personalized and precision medicine.**
